# Supplementary figures and images for: ICOR: improving codon optimization with recurrent neural networks
Source: BMC Bioinformatics. 2023 Apr 4;24:132. doi: 10.1186/s12859-023-05246-8 (PMC10074884; doi:10.1186/s12859-023-05246-8)

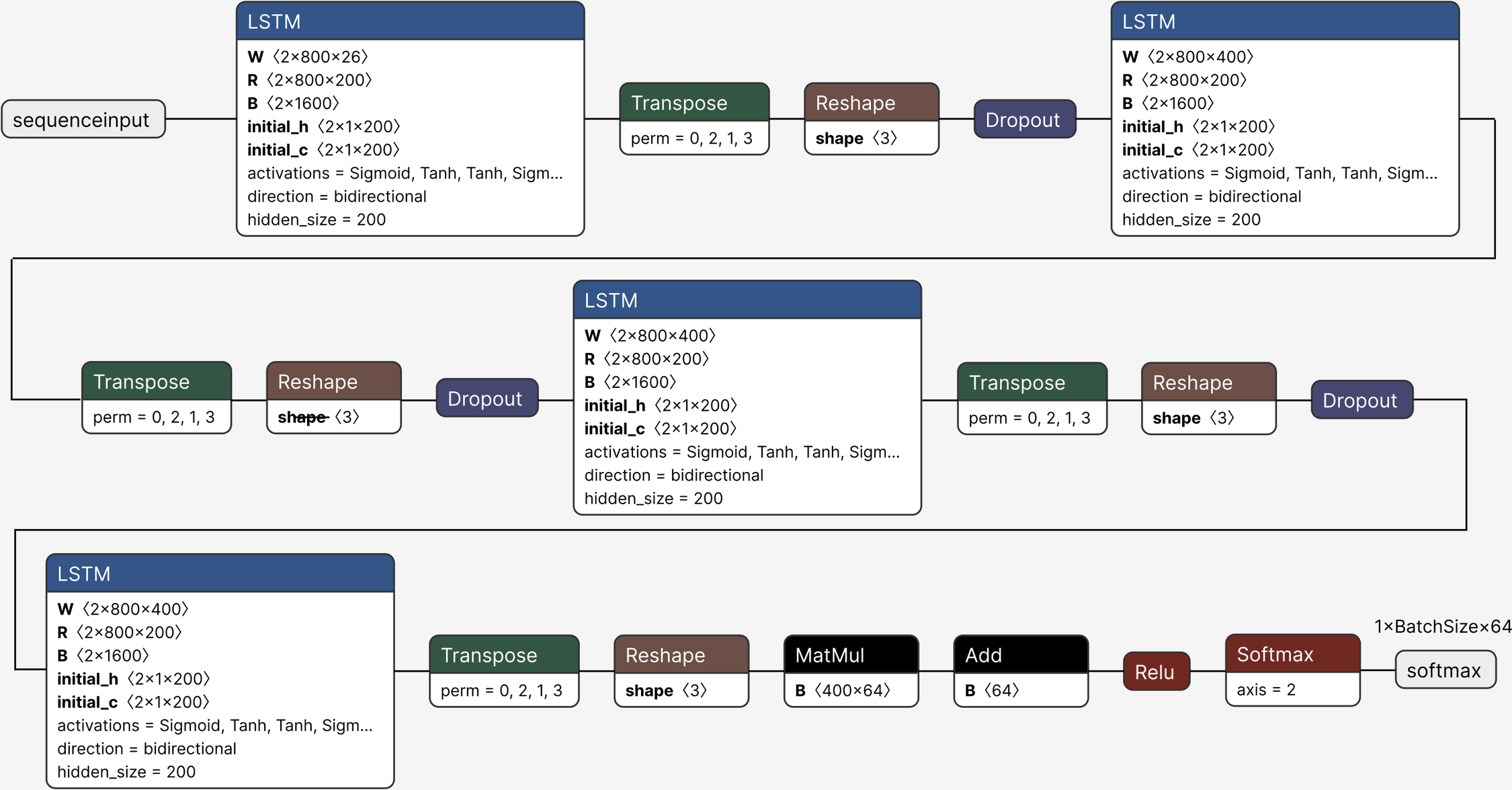

Supplement: Supplementary file 2 — Additional file 2. Figure S1 ICOR model architecture. [file 12859_2023_5246_MOESM2_ESM.tif]

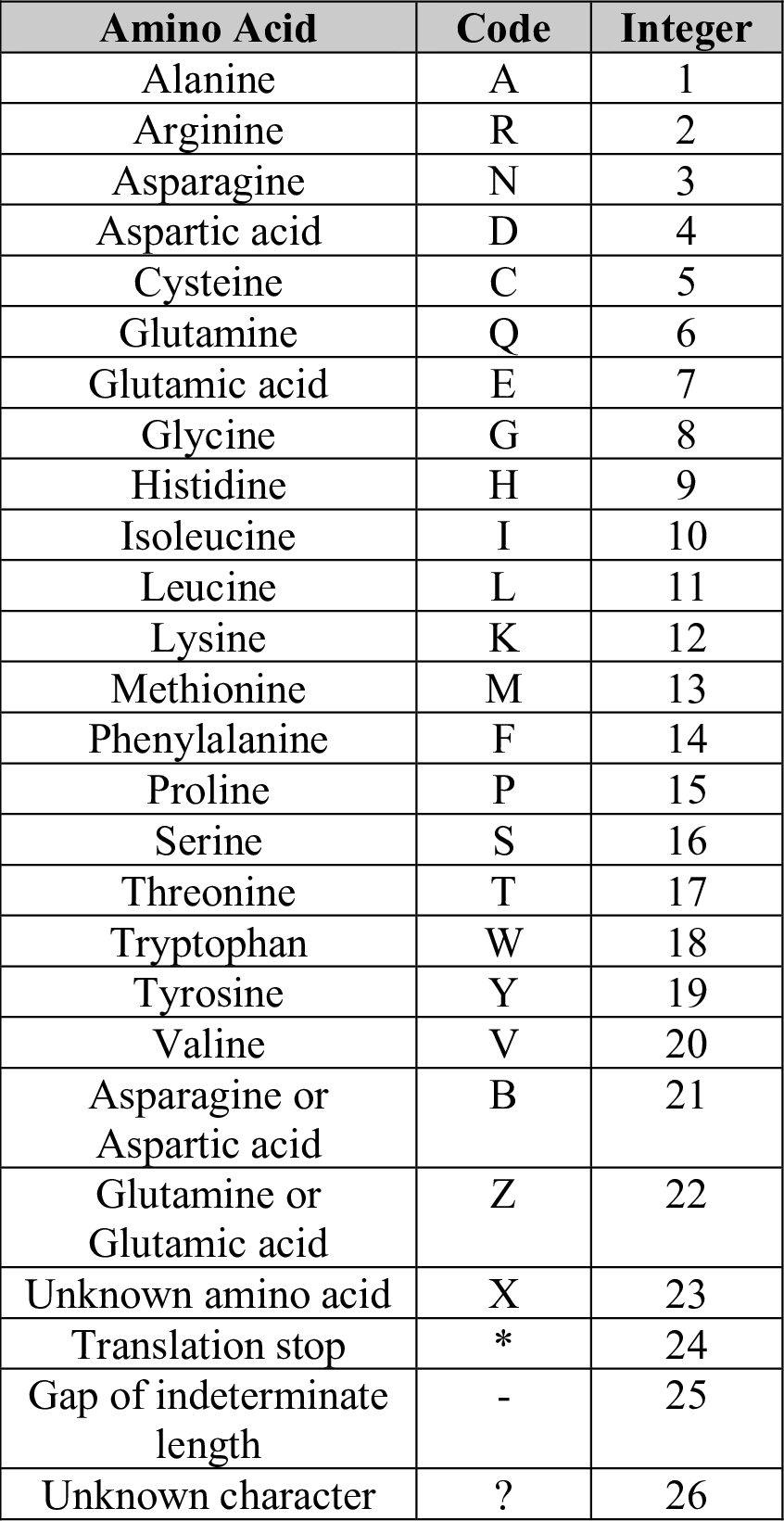

Supplement: Supplementary file 6 — Additional file 6. Amino acid table with relevant abbreviations. [file 12859_2023_5246_MOESM6_ESM.tif]
