# Supplementary material for: ICOR: improving codon optimization with recurrent neural networks
Source: BMC Bioinformatics. 2023 Apr 4;24:132. doi: 10.1186/s12859-023-05246-8 (PMC10074884; doi:10.1186/s12859-023-05246-8)
Supplement: Supplementary file 5 — Additional file 5. S2 Appendix describes the training dataset specifications. [file 12859_2023_5246_MOESM5_ESM.docx]

**Training Data Specifications**


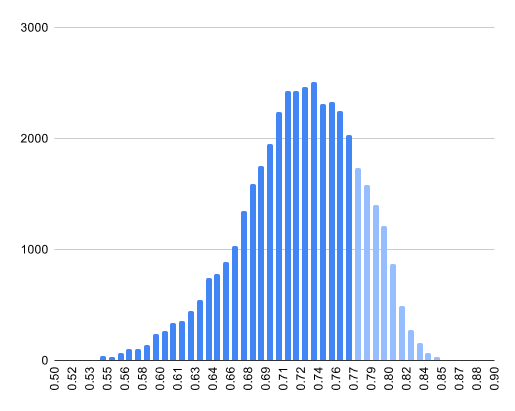


This histogram displays the distribution of the codon adaptation index of the 42,266 genes utilized in the ICOR training dataset. Codon adaptation index (CAI) is depicted on the x-axis and proportion of the overall dataset is depicted on the y-axis. Genes utilized in the final training dataset are highlighted with a lighter color.

The 7,406 genes (corresponding to the 82nd percentile) with the highest CAI were selected to use as part of the training dataset. We intentionally choose to train only on *E. coli* genes because they will be stable within the chassis during expression. Using these genes to inform codon selection in a synthetic gene may bring about this stability. Because the model only predicts based on genes from *E. coli*, based on evolutionary-instilled processes, these genes naturally expressed will theoretically not be insoluble. However, these genes vary in expression levels.
