# Supplementary material for: ICOR: improving codon optimization with recurrent neural networks
Source: BMC Bioinformatics. 2023 Apr 4;24:132. doi: 10.1186/s12859-023-05246-8 (PMC10074884; doi:10.1186/s12859-023-05246-8)
Supplement: Supplementary file 7 — Additional file 7. Table S2 describes the training hyperparameters and values. [file 12859_2023_5246_MOESM7_ESM.pdf]

Hyperparameter Used in Model Training	Value of Parameter
Gradient Decay Factor (dictates the decay rate of gradient moving average)	0.95
Learning Rate (the extent to which weights are updated during training, also known as step size)	5×10^{-5}
L2 Regularization (factor of weight decay)	1e-8
Shuffling (shuffles the order of training data)	never
Dropout (the factor by which neurons are temporarily ignored during training)	0.2
Padding (ensures a ragged input—sequences are not padded to a uniform length)	off
Sorting (sort input by ascending sequence length)	on
Optimizer (the solver algorithm used when training the network)	adam (adaptive moment estimation)
